# Application of resveratrol on oxidative stability of protein-based Antarctic krill oil high internal phase emulsion

**DOI:** 10.1016/j.fochx.2024.101727

**Published:** 2024-08-13

**Authors:** Yang Li, Xuening Yu, Rui Liu, Xiaoyang Liu, Fawen Yin, Deyang Li, Pengfei Jiang, Dayong Zhou

**Affiliations:** SKL of Marine Food Processing & Safety Control, National Engineering Research Center of Seafood, Collaborative Innovation Center of Seafood Deep Processing, Liaoning Province Key Laboratory for Marine Food Science and Technology, School of Food Science and Technology, Dalian Polytechnic University, Dalian 116034, China

**Keywords:** RES, Protein, Antioxidant, HIPEs, digestion

## Abstract

Antarctic krill oil (KO) is known for its poor oxidative stability, especially in emulsion systems. In this experiment, a complex of scallop water-soluble protein-resveratrol (SWPs-RES) was mixed with KO to create high internal phase emulsions (HIPEs) with varying RES ratios. The addition of RES led to noticeable conformational changes in SWPs, including fluorescence bursts, alterations in secondary structure, and modifications in binding motifs. The SWPs-RES complex (1:0.2) demonstrated the most effective free radical scavenging activities (HO: 38.61%, DPPH: 72.49%, ABTS: 85.66%), while the SWPs-RES complex (1:0.025) exhibited the highest emulsifying capacity. Furthermore, HIPEs containing the SWPs-RES complex (1:0.2) displayed improved rheological properties, physical stability, and enhanced oxidative stability against lipid oxidation during storage and simulated *in vitro* digestion. This study lays a scientific foundation for the utilization of scallop protein and Antarctic krill oil in the food industry.

## Highlights

• SWPS-RES complexes possessed good antioxidant and emulsifying properties
• HIPEs of SWPs-RES-KO showed better physical and oxidative stability
• HIPEs of SWPs-RES-KO showed better antioxidant capacity during storage
• HIPEs of SWPs-RES-KO exhibited well oxidative stability during digestion

## Graphical abstract

**Figure:**
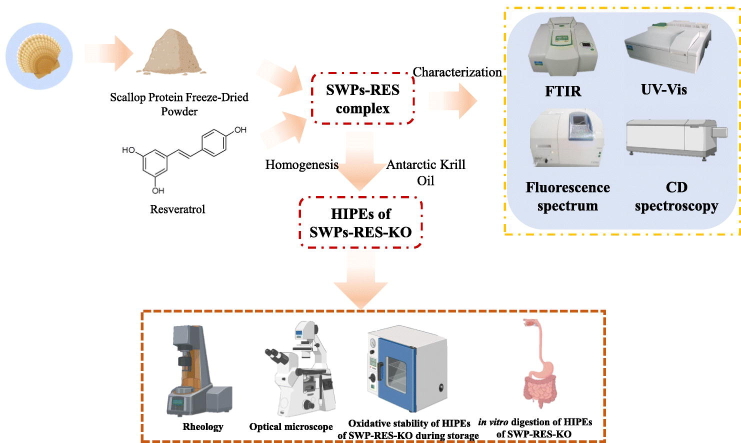

## 1 Introduction

High internal phase emulsions (HIPEs) are a kind of emulsions that contain dispersed phase volume fraction of >74% (Samanta, Nandan, & Srivastava, 2016), which is always to be called the super concentrated emulsions. Among all the types of HIPEs, three main types are frequently included and applied, such as the water-in-oil emulsion system, oil-in-water emulsion system, and supercritical CO_2_ (Zhu, 2019). In the food and cosmetics industries, there has been a growing interest in the applications of HIPEs in both research and potential commercial value in recent years. Emulsions containing unsaturated fatty acids have been found to have poor oxidative stability, making them prone to oxidation. This can lead to the accumulation of hydrogen peroxide, a lipid oxidation product, at the oil-water interface in the emulsions, further exacerbating lipid oxidation reactions (Mcclements & Decker, 2000). Therefore, to develop stable and healthy HIPEs with polyunsaturated fatty acid, it is crucial to reduce or inhibit lipid oxidation (Li et al., 2020).

The use of natural polyphenols to inhibit lipid oxidation has become a focus of researchers due to their well-known health benefits and strong antioxidant properties (Brewer, 2011). The non-flavonoid polyphenol complex, trans-3,5,40-trihydroxystilbene (resveratrol, RES), is found in various plants like grapes, tiger nuts, peanuts, blueberries, mulberry fruits, and cassia seeds, existing in both cis and trans configurations (Yang et al., 2019). RES has been utilized as an antioxidant in food industry applications and experimental research due to its remarkable antioxidant properties (El-Maksoud et al., 2018). However, the limited water solubility and absence at the interface of oil-in-water emulsion systems hinder the maximum utilization of RES's antioxidant effects in complex food-associated liquid systems (Mickaël et al., 2009). Therefore, to address this issue, modifying RES through interactions with amphoteric substances can facilitate the relocation of RES molecules to the emulsion system interface, thereby enhancing their antioxidant effects in emulsions. For example, soy protein-resveratrol complexes have been employed as emulsifiers to improve the antioxidant functionality of RES in oil-in-water emulsions (Cheng, Fang, Wusigale Bakry, Chen, & Liang, 2018).

Food-derived proteins, such as those from scallops, are commonly utilized to enhance the bioavailability, stability, and water solubility of resveratrol (RES). Numerous studies have explored the use of natural proteins conjugated with RES to improve its utilization (Wan, Wang, Wang, Yuan, & Yang, 2014). The interaction between polyphenols and proteins involves various non-covalent bonds, including hydrophobic, ionic, and hydrogen bonds, as well as covalent bonds (You, Luo, & Wu, 2014). However, covalent complexes formed through covalent bonding are preferred due to their stronger, more enduring interactions, and greater stability (Liu, Ma, Gao, & McClements, 2017). Antarctic krill oil is rich in the unsaturated fatty acids, astaxanthin and other functional components, which is highly susceptible to light and temperature and other external environmental factors, resulting the sensitive oxidative deterioration of Antarctic krill oil during the storage process (Zheng, Zhu, & Dai, 2021). The phospholipids is proved to be rich in Antarctic oil but its characteristics of water expansion and cohesion induce the poor water solubility of phospholipids, highly affecting the commercial value of Antarctic krill oil and the food safety (Lu, Bruheim, & Jacobsen, 2014).

In this study, the interaction between RES and SWPs in different ratios in the KO-HIPEs system and their oxidative stability were investigated. The conformational information of SWPs and SWPs-RES complexes were analyzed using circular dichroism (CD), UV–visible spectroscopy, fluorescence spectroscopy and Fourier transform infrared spectroscopy (FTIR). The emulsification properties of the complexes were determined in determination of the value of EAI and ESI. The effect of the complexes on emulsion storage stability, rheological properties and *in vitro* digestion properties were also determined. The primary objective was to comprehend how SWPs-RES complexes influence the physicochemical stability of KO-HIPEs and to establish a theoretical foundation for the antioxidant applications in emulsification systems and *in vitro*.

## 2 Materials and methods

### 2.1 Materials

Fresh scallop was purchased from local seafood markets in Dalian (China). Resveratrol (RES) purchased from McLean (Shanghai, China). Antarctic krill oil was purchased from Liao Fishery Group Co. Ltd. (Dalian, China). DPPH and ABTS standards were purchased from Shanghai McLean Biochemical Co. Ltd. (Shanghai, China).

### 2.2 Preparation of the SWPs

Scallop water-soluble protein was prepared by alkali dissolution method. Firstly, the fresh scallop meat was minced and mixed with the distilled water at the material-liquid ratio (1:2, *w*/*v*). Then, the pH level of solution was adjusted to 11 by adding the NaOH solution (1 M) and stirred in the ice bath (4 °C, 30 min), followed by centrifugation (10,000 ×*g*, 15 min). The collected supernatant was then neutralized to a pH of 7 with HCl. Following lyophilization, the SWPs powder was obtained and stored at −20 °C for future use.

### 2.3 Preparation of the SWPs-RES complex

The method was based on previous studie (Joye, Davidov-Pardo, Ludescher, & McClements, 2015) and slightly modified. The SWPs powder was dissolved to create the protein solution with concentration of 10 mg/mL and pH level of 9. Similarly, the RES powder was dissolved in 70% ethanol-water solution to obtain RES solutions with varying concentrations (0.625, 1.25, 2.5, 5, and 10 mg/mL). The SWPs solution and RES solution were mixed in 5:1 volume ratio and incubated in a water bath at 25 °C for 2 h. This process resulted in OWPs-RES complex solutions with ratios of 1:0.0125, 1:0.025, 1:0.05, 1:0.1, and 1:0.2. Subsequently, the complex solutions were dialyzed with deionized water for 48 h, with water changes every 12 h, leading to the final SWPs-RES complex.

### 2.4 Determination of total phenolic contents of SWPs-RES complex

The RES content was analyzed using the forintol reagent method (Hu et al., 2016). The dissolved sample (0.5 mL, protein concentration 4 mg/mL) was combined with freshly prepared Folin-Ciocalteu reagent (2.5 mL, 0.2 M) in the dark for 5 mins. Subsequently, Na_2_CO_3_ solution (2 mL, 7.5%, *w*/*v*) was introduced and left to react for 2 h. The absorbance of the resulting solution was measured at 760 nm, with the protein solution serving as the blank control.

### 2.5 Fluorescence spectroscopy (FL)

The methodology was based on previous studie (Roy et al., 2012) with slight modifications. Deionized water was used to prepare the SWPs-RES complex sample to be tested (SWPs concentration was 1 mg/mL). The fluorescence singals of SWPs and SWPs-RES complex was determined by the fluorescence spectrophotometer (HitachiF-7000, Tokyo, Japan). The excitation wavelength was set at 280 nm and the emission wavelength range was set at 300–500 nm, the slit width of both was 5 nm, and the change of fluorescence intensity was recorded.

### 2.6 UV–vis spectroscopy

The solution of SWPs and SWPs-RES complex (0.2 mg/mL) were recorded by the UV–visible spectrometer (Lambda 35, PerkinElmer, Waltham, Massachusetts, USA) around the wavelength of 200–400 nm, respectively.

### 2.7 Fourier transform infrared spectroscopy (FTIR)

The SWPs and SWPs-RES complex were combined with dried spectroscopic grade KBr at a ratio of 1:100 by mass and uniformly powdered using an onyx mortar under infrared lamp irradiation. Subsequently, the samples were analyzed using FTIR (Frontier, PerkinElmer, Waltham, Massachusetts, USA).

### 2.8 Circular dichroism (CD)

The CD in the far-ultraviolet region was used to determine the secondary structure changes of protein samples. The CD analyzer (J-1500, JASCO, Japan) was used to scan in the range of 190–280 nm. The cuvette optical range, scanning rate, and the resolution were set in 1 mm, 100 nm/min, and 0.5 nm, respectively.

### 2.9 Hydroxyl free radical scavenging activity

The sample (1 mL, 1.5 mg/mL) was mixed with 1,10-phenanthroline monohydrate (1 mL, 1.865 mM), the FeSO_4_ (1 mL, 1.865 mM) and H_2_O_2_ (1 mL, 20 mM) while the whole reaction system was incubated for for 1 h at 37 °C. Then, the absorbance value was read at 536 nm.

$$\text{Hydroxyl free radical scavenging activity}\;\left(\%\right)=\left(A–\text{An}\right)/\left(\mathrm{Ab}–\text{An}\right)\times 100$$

where A was OD value of the sample solution, Ab was OD value of the solution without added H_2_O_2_, and An was OD value of the solution without added sample.

### 2.10 ABTS free radical scavenging activity

Potassium persulfate (2.4 mM) and ABTS (7 mM) solutions were mixed in a 1:1 volume ratio and the mixture was placed in storage in the dark for 12–16 h to produce ABTS. The sample solution of SWPs and SWPs-RES complex (0.5 mL, protein concentration was 0.5 mg/mL) was then dissolved in 70% ethanol aqueous solution and later added to diluted ABTS solution (2 mL) and reacted in the dark for 1 h. The absorbance value of the solution to be measured was determined at 734 nm.

$$\text{ABTS free radical scavenging activity}\;\left(\%\right)=\left(1–\mathrm{As}/\mathrm{Ac}\right)\times 100$$

where As was OD value of the test samples and Ac was OD value of the control.

### 2.11 DPPH free radical scavenging activity

The sample of SWPs and SWPs-RES complex (2 mL 1.5 mg/mL) were mixed with DPPH solution (2 mL, 0.2 mM, dissolved in 95% ethanol) and then reacted in the dark for 30 min, and then the OD value of the mixture at 517 nm was measured.

$$\text{DPPH free radical scavenging activity}\;\left(\%\right)=1-\left(\mathrm{As}▬\mathrm{Ac}\right)/\mathrm{Ab}\times 100$$

where Ab was OD value of the solution without sample addition, As was OD value of the solution after sample addition, Ac was OD value of the sample.

### 2.12 Determination of emulsifying properties of SWPs-RES complex

The emulsion system was prepared by mixed of SWPs and SWPs-RES complex (15 mL, 2 mg/mL) and 5 mL of soybean oil, and then homogenized twice for 1 min each time using the homogenizer at 10,000 rpm (T25 Digital, IKA, Staufen, Germany). 0.05 mL of the homogenized emulsion was diluted 100-fold with SDS solution (0.1%, *w*/*v*) and mixed well, and SDS solution (0.1%) was used as the control, and then the absorbance value was measured immediately at 500 nm.

$$\mathrm{EAI}\;\left(m^{2}/g\right)=\left(2\times 2.303\times A_{500}\times \mathrm{dil}\right)/1000\text{0LφC}$$

where A_500_ was the OD value at 500 nm, dil was the dilution factor (100), L was passage path length of the cuvette (m), C was the protein concentration in the solution (g/m^3^), and φ was the volume fraction of the oil phase (0.25).

$$\mathrm{ESI}\;\left(\min\right)=\left(A_{0}/A_{0}–A_{15}\right)\times \text{∆t}$$

where A_0_ and A_15_ were the OD value of thesample at 0 min and 10 min after homogenization, ∆t was the resting time (15 min).

### 2.13 Preparation of the HIPEs of SWPs-RES-KO

KO-HIPEs stabilized by SWPs and SWPs-RES was prepared by a previous method (Bi et al., 2020). KO was first diluted with soybean oil (KO content of 15%, m/m). The oil phase, SWPs or SWPs-RES complex, and Tween-20 were mixed in a mass ratio of 1:0.2:0.04, and then the mixtures were homogenized using a digital Ultra-Turrax mixer (T25, IKA, Staufen, Germany) at 10,000 rpm for 1.5 min to obtain oil-to-mass ratios (φ) of 80% for the HIPEs.

### 2.14 Determination of rheological properties of HIPEs of SWPs-RES-KO

The rheology of HIPEs containing SWPs-RES-KO was analyzed using a rheometer (Discovery HR-1, TA Instruments, New Castle, DE, US). Frequency scan measurements were conducted across a range of 0.1 to 100 Hz to observe the storage and loss moduli (G' and G") of the HIPE. Furthermore, shear scans were carried out from 0.1 to 100/s to study the impact of shear rate on the apparent viscosity (η) of the HIPEs.

### 2.15 Determination of oxidative stability of HIPEs in storage

Freshly prepared emulsions were stored at a constant temperature of 40 °C and samples of HIPEs were taken every 1 day to extract oil to assess lipid oxidation.

#### 2.15.1 Determination of peroxide value

The methodology is based on previous studie (Abuzaytoun, Budge, Hansen, & MacKinnon, 2020) with minor modifications. It was described as follows: 0.01 g of oil was fully dissolved in 1.5 mL of a mixture of dichloromethane and 95% ethanol (volume ratio: 3:2). Then, the ammonium hexahydrate-ferric sulfate (5 mM, 100 μL), 0.25 M methanol‑sulfuric acid (200 μL), and 1 M methanol-dimethylphenol orange tetrasodium salt (200 μL) were added and mixed well. After incubation for 30 min in the dark, pure water (1 mL) was added to the reaction system. It was then centrifuged for 5 min at 4000 *g*. The upper layer of the mixture (200 μL) was collected while the absorbance was measured at 560 nm. The POV value (mg/kg oil) was calculated after plotting the standard curve for isopropyl benzene hydroperoxide.

#### 2.15.2 Determination of thiobarbituric acid value

The methodology was based on previous study (John et al., 2005) with slight modifications. The 0.1 g oil was homogeneously mixed with a solution (2.5 mL) containing hydrochloric acid (4.17 mL), distilled water (196 mL), thiobarbituric acid (0.75 g), and trichloroacetic acid (30 g). This mixture was then incubated in boiling water for 10 mins. After cooling, the mixture was centrifuged at 3000 *g* for 10 mins, and the optical density (OD) value of the upper layer (200 μL) was measured at 532 nm. The concentrations of malondialdehyde were then converted to TBARS values using a specific formula:

$$\text{TBARS}\;\left(\mathrm{mg}/\mathrm{kg}\;\text{oil}\right)=A_{532}\times 2.77$$

where A_532_ was the OD value at 532 nm.

### 2.16 Determination of *in vitro* oxidative stability of HIPEs during GI digestion

The method for simulating *in vitro* digestion was referenced from previous studies with some modifications (Han et al., 2019). The artificial saliva was pre-warmed to 37 °C and then added to each HIPEs (7.5 g) in a quantity of 7.5 mL. The mixture underwent incubation at 37 °C on a constant temperature shaker for 10 mins to mimic oral digestion. Subsequently, 15 mL of gastric fluid (SGF, pH = 2.5) was introduced to the oral digestion samples, with pH adjustment to 2.5, and further incubated for 2 h to imitate gastric digestion. Following this, 1.5 mL of simulated intestinal fluid (SIF) was combined with the stomach digested samples, adjusting the pH to 7.0. Bile salts (53.6 mg/mL), trypsin (24 mg/mL), and lipase (24 mg/mL) were then added in quantities of 3.5 mL, 2.5 mL, and 2.5 mL, respectively. The mixture was agitated at 37 °C for 2 h to replicate the intestinal environment. The solution post-simulated intestinal digestion was collected to assess the oxidation of fats and oils pre and post emulsion digestion.

### 2.17 Statistical analysis

The study was carried out three times, with the results presented as mean ± standard deviation. Tukey's test was utilized for statistical comparisons between groups using SPSS 19 software (SPSS Inc., Chicago, IL, USA). Statistical significance was defined as *P* < 0.05.

## 3 Results and discussion

### 3.1 Characterization of the structures of SWPs and SWPs-RES complex

**Fig. 1:**
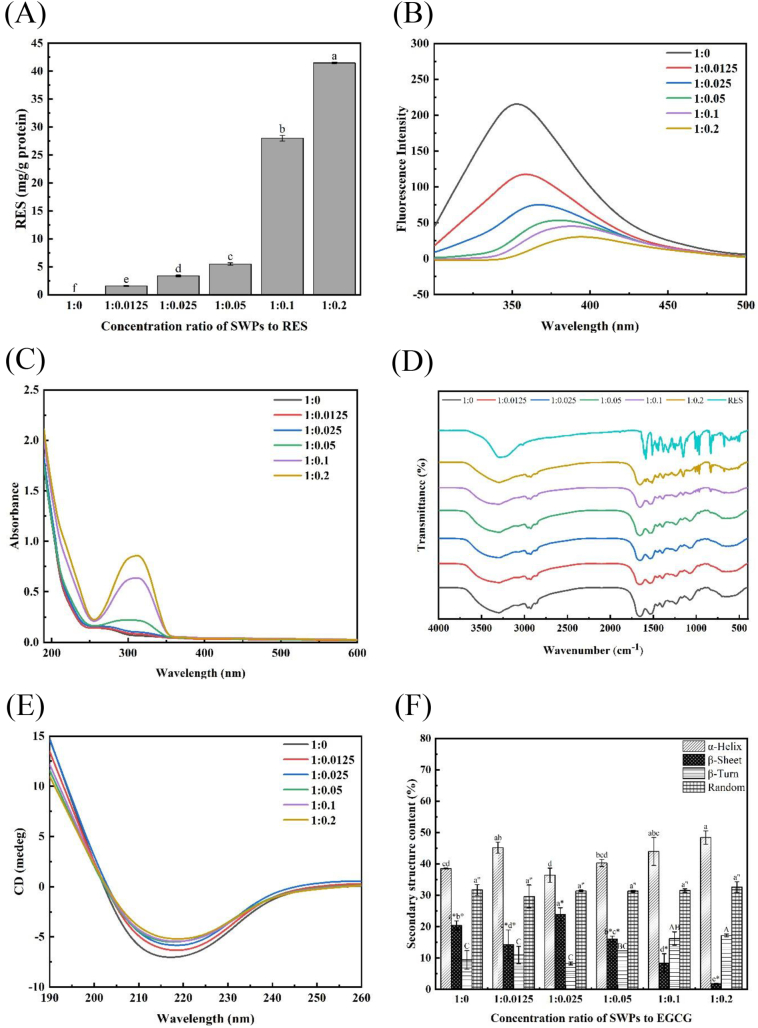
(A) Phenol content in SWPs and SWPs-RES. (B) Endogenous fluorescence spectra of SWPs and SWPs-RES complex. (C) UV–vis spectra of SWPs and SWPs-RES complex. (D) Fourier transform infrared spectra of SWPs and SWPs-RES complex. (E) Circular dichroism of SWPs and SWPs-RES complex. (F) Secondary structure diagrams of SWPs and SWPs-RES complex. A-F represented the different ratio of SWPs to RES (1:0, 1:0.0125, 1:0.025, 1:0.05, 1:0.1, 1:0.2).

The interaction between polyphenols and proteins can result in conformational changes of the protein, affecting fluorescence function, secondary structures, and specific bonds. These changes can be assessed using various experimental instruments such as fluorescence spectroscopy, UV–Vis spectroscopy, FTIR, and CD spectrum. To determine the loading amounts of RES on SWPs-RES complex, the phenol contents in the complexes were first characterized after removing free RES molecules through dialysis. The results in Fig. 1A indicated the significant increase in phenol contents in the complexes in a ratio-dependent manner, eventually exceeding 40 mg/g of proteins. It was evident that the accumulation of RES in the SWPs-RES complex substantially increased from ratio of 1:0.1 to 1:0.2, while the SEPs-RES complex at lower ratios showed slower increases. Moreover, the enhancement at ratio of 1:0.2 appeared to be less than that at ratio of 1:0.1, suggesting that the addition of RES at ratio of 1:0.2 might be approaching the loading limit of SWPs at these concentrations. Some previous studies have combined RES with proteins to form the protein-RES complexes, including RES-soy protein complexes (Wan et al., 2014), RES-covalent complexes with zeinolysin (Ren et al., 2022), and RES-high-phosphate-yolk protein complexes (Duan et al., 2016). The enhanced oxidative stability of corn oil/water emulsions stabilized by SPI-RES complexes was examined through the preparation of these complexes. This improvement can be credited to the specific enrichment of RES at the interface of oil and water, alongside the adsorption of SPI. As a result, the molecules of RES are driven towards the oil/water interface in the emulsion by the increased hydrophobicity introduced by the SWPs.

Fluorescence spectroscopy was utilized to investigate the conformational changes of SWPs upon binding with RES, as depicted in Fig. 1B. The fluorescence intensity of SWPs was significantly higher compared to the SWPs-RES complex. Moreover, the fluorescence intensity decreased progressively with increasing RES concentration, indicating the quenching properties of RES on the intrinsic fluorescence of SWPs, in line with previous research (Hu et al., 2023). Furthermore, under an excitation wavelength of 280 nm, the maximum emission wavelength of SWPs was 350 nm. As the ratios of RES contents increased, the maximum emission wavelength of the SWPs-RES complex shifted from 353 nm to 394 nm, suggesting that the tryptophan (Trp) residue, the primary fluorophore in the proteins, might have been exposed to more hydrophilic environment, leading to the observed red-shift in signals (Wan et al., 2014). These results indicated that the binding of RES to SWPs induced specific conformational changes in SWPs, providing further evidence of potential interaction between RES and the amino acid residues of the fluorophore in SWPs.

UV spectroscopy was used to characterize the structural changes of proteins in SWPs and SWPs-RES complex. The structural changes of proteins in SWPs and SWPs-RES complex were characterized and the results were shown in Fig. 1C. Among all the samples, the major absorption peaks were discovered at approximately 280 nm, which was primarily caused by the aromatic heterocycles of tyrosine and tryptophan residues of scallop proteins. The absorption intensity of SWPs-RES complex was significantly higher than that of SWPs with the red-shift in the maximum absorption wavelength. The reason for this phenomenon might be due to the binding of SWPs to RES, which altered the structure of the proteins and triggered more exposure of tyrosine and tryptophan residues to the surrounding environment (Han et al., 2019). On the other hand, it was reported from previous studies (Dai et al., 2019) that the RES showed the significant absorption peak at approximately 300 nm, possibly explaining the reason why the SWPs-RES complex exhibited enhancement of absorption intensity as well as the emerge of redshift. This result was consistent with the results of UV–Vis absorption spectra of zeinolysin-RES complexes in previous reports (Ren et al., 2022).

FTIR analysis was conducted to investigate changes in molecular structure and interactions between RES and SWPs. Peaks corresponding to N—H and C—H bonds stretching vibrations in the SWPs group were observed at 3298 cm^−1^ and 2928 cm^−1^, as shown in Fig. 1D. Additionally, absorption peaks at 1657 cm^−1^ (C—O bond stretching vibration) and 1535 cm^−1^ (N—H bond bending vibration and C—N bond stretching vibration) were identified, representing the amide I and II bands of SWPs (Han et al., 2019). The signals of SWPs-RES complexes showed a slight shift in the peak near 3300 cm^−1^ compared to SWPs alone, attributed to the hydroxide group stretching vibration (Cerqueira, Souza, Teixeira, & Vicente, 2011). Changes in the peaks of amide I and amide II bands of SWPs-RES complexes indicated alterations in the protein's secondary structure upon binding with SWPs. Moreover, these findings suggested the occurrence of hydrogen bonding during the formation of the OWP-EGCG complex, aligning with previous research on the interaction between zeaxanthin and RES (Ren et al., 2022).

Finally, The CD spectrum was utilized to analyze the alterations in protein secondary structure in both SWPs and SWPs-RES complex. In Fig. 1E&1F, the CD spectra of SWPs indicated the gradual increase in the characteristic peak (negative value) associated with α-helix recognition at around 215 nm with the progressive addition of RES, aligning with previous research (Yi, Fan, Zhang, & Zhao, 2016). The secondary structure composition of SWPs included α-helix (38.5%), β-sheet (20.4%), β-turn (9.4%), and random coil (31.7%), whereas the SWPs-RES complexes showed an increasing trend in α-helix and β-turn content. Notably, the β-sheet content exhibited the decreasing trend proportionate to the ratio, reaching a minimum level at a ratio of 1:02 (1.85%). The interaction between RES and SWPs led to significant changes in the secondary conformation of SWPs, possibly influenced by hydrophobic and hydrogen-bonding interactions resulting in structural alterations. Moreover, the ratio-dependent increase in α-helix content was observed, suggesting that non-covalent interactions between SWPs and RES played a more prominent role compared to covalent interactions (Wang, Li, Hou, Zhang, & Li, 2023). Furthermore, we investigated the particle size and morphological characteristics of the SWP-RES complex at different ratios, as determined by laser particle sizing and scanning electron microscopy (SEM) (Fig. S1 & S2). The results indicated that the particle size of the SWP-RES complex exhibited an initial increase followed by a decrease, with the 1:0.5 ratio yielding the smallest particle size. The reduced particle size of the SWP-RES complex (1:0.5) contributed to its uniform distribution at the oil/water interface in the emulsion system, enhancing its emulsifying capacity (consistent with the emulsifying activity index (EAI) results shown in Fig. 3A), which may have led to the relatively smaller emulsion droplet size (Fig. 4D). The comparison of the SEM images across different ratios of the SWP-RES complex revealed that the SWP complex displayed non-uniform appearances and the lamellar structure. The incorporation of RES significantly enhanced the crosslinking structure of the SWPs, which aligns with interactions observed between proteins and other polyphenols, as noted in the study by Li et al. (2023).

### 3.2 Free radical scavenging capacity of SWPs and SWPs-RES complex

**Fig. 2:**
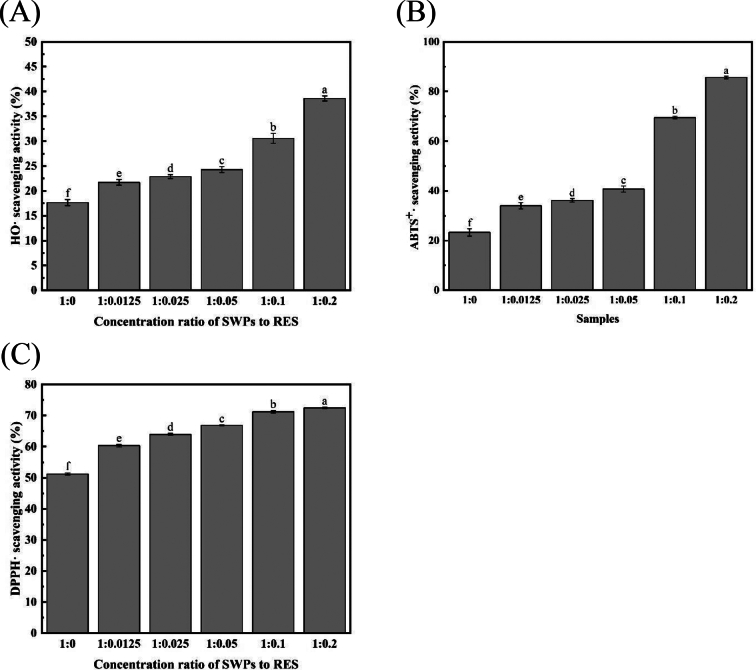
(A) OH radical scavenging capacity of SWPs and SWPs-RES complex. (B) ABTS radical scavenging capacity of SWPs and SWPs-RES complex. (C) DPPH radical scavenging capacity of SWPs and SWPs-RES complex. A-F represented the different ratio of SWPs to RES (1:0, 1:0.0125, 1:0.025, 1:0.05, 1:0.1, 1:0.2).

The study evaluated the antioxidant capacity of SWPs and SWPS-RES complex by measuring their ability to scavenge free radicals such as hydroxyl, ABTS, and DPPH. Hydroxyl free radicals play a crucial role in both oxidative processes and lipid oxidation initiation. The results depicted in Fig. 2A showed that the hydroxyl free radicals scavenging capacity increased linearly with higher RES content, reaching its peak (38.61%) at a SWPs to RES ratio of 1:0.2. This enhancement could be attributed to the improved HO scavenging ability of the protein when bound to RES, indicating the superior antioxidant capacity of the SWPs-RES complex. Hydroxyl radicals are water-soluble radicals, and the enhanced ability of the SWPs-RES complexes to scavenge hydroxyl radicals suggested that the antioxidant capacity of the SWPs-RES complexes was effective in the water-soluble environment (Liu et al., 2018). The ABTS free radicals scavenging capacity of SWPs and SWPs-RES complex was investigated in Fig. 2B. The results indicated that SWPs exhibited the lowest ABTS free radicals scavenging capacity, with values gradually increasing in a ratio-dependent manner of RES. The highest ABTS free radicals scavenging ability (85.65%) was observed at a SWPs to RES ratio of 1:0.2, significantly higher than other ratios of the SWPs-RES complex. This may be attributed to the increased antioxidant properties when more RES is bound to SWPs. The antioxidant efficiency is influenced by the number and location of hydroxyl groups, with resveratrol containing three hydroxyl groups in its benzyl portion, enhancing its free radical scavenging abilities (Kotora et al., 2016). Additionally, the DPPH free radicals scavenging capacity assay was conducted, as depicted in Fig. 2C. The DPPH free radicals scavenging capacity showed a linear increase with higher proportions of RES. SWPs exhibited the lowest DPPH free radicals scavenging capacity, while the highest value of 72.49% was observed at a SWPs to RES ratio of 1:0.2, consistent with the results from the ABTS assay. The antioxidant effect of the SWPs-RES complex in a hydrophobic system was demonstrated through its DPPH free radical scavenging activity, as DPPH free radicals are more soluble in hydrophobic environments (Li et al., 2023).

### 3.3 EAI and ESI of SWPs and SWPs-RES complexes

**Fig. 3:**
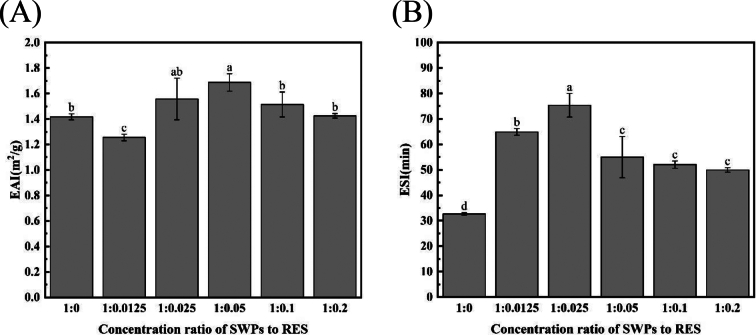
(A) Emulsifying activity index of SWPs and SWPs-RES complex. (B) Emulsification stability index of SWPs and SWPs-RES complex. A-F represented the different ratio of SWPs to RES (1:0, 1:0.0125, 1:0.025, 1:0.05, 1:0.1, 1:0.2).

In this study, the emulsification properties of SWPs and SWPs-RES complex were evaluated by determinations of values of EAI and ESI, and the results were shown in Fig. 3. As shown in Fig. 3A, the EAI value of SWPs-RES complex showed the trend of first increasing and then decreasing by additions of RES onto SWPs while the EAI of the SWPs-RES complex reached the maximum level of 55.03 m^2^/g at the ratio (1:0.05) of SWPs to RES, suggesting that interaction between SWPs and RES under this ratio contributed to the formation that possessed the highest emulsifying ability with Antarctic krill oil. The change in the value of EAI was attributed to the facts that the interactions between SWPs and RES affected the emulsifying ability of proteins (Deng et al., 2023), and the proper contents of RES may alter the flexibility, solubility and surface hydrophobicity of proteins, thus altering the molecular conformation of SWPs as well as their emulsification properties (Li et al., 2020).

As shown in Fig. 3B, the ESI of SWPs-RES complex exhibited the similar tendency that the value firstly increased and then decreased in a ratio-dependent manner of RES, finally reaching the maximum level of 1.56 min at the ratio of SWPs to RES of 1:0.025. The addition of RES increased the ESI of the SWPs-RES complex, which was consistent with the previous studies (Li et al., 2023). The corresponding reasons might be due to the fact that the introduction of polyphenols altered the interactions between the proteins, which increased the site resistance and electrostatic repulsion between the droplets to a certain extent, thus decreasing the interfacial tension at the oil-water interface and preventing the aggregation of droplets (Wang et al., 2023).

### 3.4 Rheological properties of HIPEs of SWPs-RES-KO

**Fig. 4:**
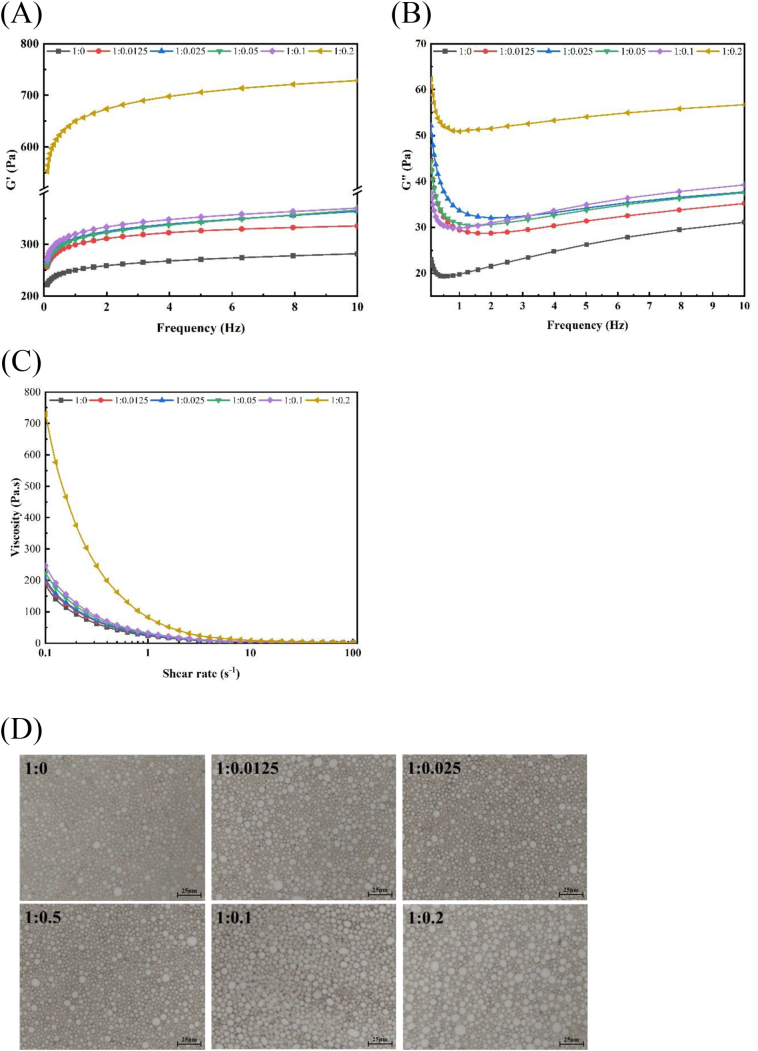
(A) G' of the KO-HIPEs stabilized by SWPs and SWPs-RES complex. (B) G" of the KO-HIPEs stabilized by SWPs and SWPs-RES complex. (C) Viscosity of the KO-HIPEs stabilized by SWPs and SWPs-RES complex. (D) Optical micrographs of KO-HIPE stabilized by SWPs and SWPs-RES complex.

Rheological properties are considered the most critical parameters for assessing the stability and applicability of HIPEs. In this study, Antarctic krill oil was combined with SWPs-RES complex to create HIPEs of SWPs-RES-KO, and the rheological properties of these HIPEs were analyzed. The relationship between frequency and energy storage modulus (G') and loss modulus (G") in HIPEs of SWPs-RES-KO was analyzed. Results from Fig. 4A & 4B demonstrated that within the test range, all emulsions consistently exhibited higher G' values compared to G", suggesting superior elastic behavior over viscous behavior. The higher G' values indicated increased resistance to external forces and enhanced stability of the emulsions. (Liu et al., 2019). Furthermore, compared within the applications of SWPs and SWPs-RES complex, both G' and G“ values in HIPEs of SWPs-RES-KO complex were higher than HIPEs of SWPs-KO where their values both ratio-dependently increased, suggesting that the incorporation of RES improved the rheological properties of the emulsions. In addition, we also found that the values of G" and G' at ratio of 1:0.2 shared obviously high differences compared with other groups of HIPEs of SWPs-RES-KO. This was similar to previous results on the rheological characterization of HIPPEs stabilized by perch protein-polyphenol complexes (Zhang et al., 2023). In certain instances of highly internal phase emulsions (HIPEs), the system of emulsion stabilized and separated by solid particles with a higher internal phase fraction (>0.74) was identified as high internal phase Pickering emulsions (HIPPEs). Colloidal particle-stabilized emulsions (Pickering emulsions) exhibit impressive stability and anti-coalescence properties. The protein modification facilitated by polyphenol, supported by either covalent or non-covalent bonds, can enhance Pickering emulsion stability. The formation of a dense network, facilitated by hydrogen bonds between proteins and polyphenol, can impede the coalescence of oil droplets. This results in reduced droplet sizes in HIPPEs and increased stability of emulsions (Chen et al., 2023), as similarly reported in other studies (Xiong, Li, & Yang, 2023). Our research demonstrated that the inclusion of resveratrol (RES) in SWPs-RES complexes impacted the rheological properties of HIPEs. Although the SWPs-RES complex was unable to form Pickering emulsions with knockout oil (KO) in the present conditions, it was still able to maintain the physical and oxidative stability of HIPEs of KO.

The relationship between shear rate and apparent viscosity in HIPEs of SWPs-RES-KO was established in this study. Fig. 4C illustrated that the viscosity of all samples decreased with increasing shear rate, indicating shear thinning behavior and confirming the pseudoplastic nature of the fluids. The phenomenon of shear thinning could be attributed to the disruption of the internal droplet structure and rearrangement at higher shear rates, as discussed as previously reported (Wang et al., 2024). Notably, the HIPEs of SWPs-KO exhibited the lowest apparent viscosity, while the HIPEs of SWPs-RES-KO showed a ratio-dependent increase in apparent viscosity. The highest apparent viscosity was observed when the SWPs to RES ratio was 1:0.2, possibly due to the alteration of SWPs' surface electrostatic charge by RES, leading to varied apparent viscosities of the emulsions (Chen et al., 2023). Utilizing ultrasonic technology is an alternative method for enhancing the emulsification capabilities and stability of emulsion systems with environmentally friendly properties. When ultrasound waves penetrate the emulsifying layer within emulsions, cavitation bubbles rapidly form and promptly collapse, leading to alterations in temperature and pressure within the emulsifying layer. This process results in physical, chemical, and thermal effects within the emulsifying layer. As a result, the emulsifying agent is efficiently and swiftly dispersed at the interface through acoustic cavitation and shear induced by ultrasound, resulting in the formation of emulsions with fine and evenly sized droplets. This treatment is considered essential for enhancing the stability of emulsion systems. For example, emulsions of rice bran protein-chlorogenic acid subjected to ultrasound-assisted treatment demonstrated a significant enhancement in stability (Wang et al., 2022). In future studies, ultrasonic treatment remains a viable method for enhancing the stability of HIPEs of SWPs-RES-KO.

The microstructure of HIPEs of SWPs-RES-KO was observed using a fluorescence inverted microscope, as depicted in Fig. 4D. The droplet sizes of the emulsions exhibited varying changes with increasing RES concentration, indicating a modification in the protein structure due to the addition of RES. This alteration subsequently influenced the physical stability of the emulsions (Gong et al., 2020). Emulsions stabilized by the SWPs-RES complex displayed more uniform particle size, with oil droplets positioned closely to each other without polymerization, suggesting improved stability attributed to the presence of RES. Moreover, HIPEs stabilized by the SWPs-RES complex (1:0.2) showcased larger droplets, possibly because the SWPs-RES complex was adsorbed at the oil droplet interface, leading to increased particle sizes. Similar findings were reported in previous studies where zein nanoparticles were absorbed onto oil droplet surfaces in the presence of GA (Song et al., 2023). Aside from HIPEs, utilizing nano-emulsions with droplet sizes smaller than 500 nm can enhance surface area, facilitating interaction between outer environments and internally trapped oil droplets. Studies have shown that incorporating nano-emulsions can mitigate autoxidation of unsaturated fatty acids and alter fatty acid composition within emulsions (Uçar, 2020). Furthermore, nano-emulsions have been linked to gastrointestinal (GI) digestion, wherein proteins undergo initial hydrolysis during the gastric phase followed by increased hydrolysis in the small intestinal phase (Ren, Xiong, & Li, 2023). In the case of HIPEs, the abundance of internal oil makes them susceptible to autooxidation, particularly in HIPEs containing SWPs-RES-KO due to their higher phospholipid content. Therefore, altering the droplet size of HIPEs to nano-emulsions appears to be an effective approach in reducing emulsion auto-oxidation.

### 3.5 Storage stability of HIPEs of SWPs-RES-KO against lipid oxidation

**Fig. 5:**
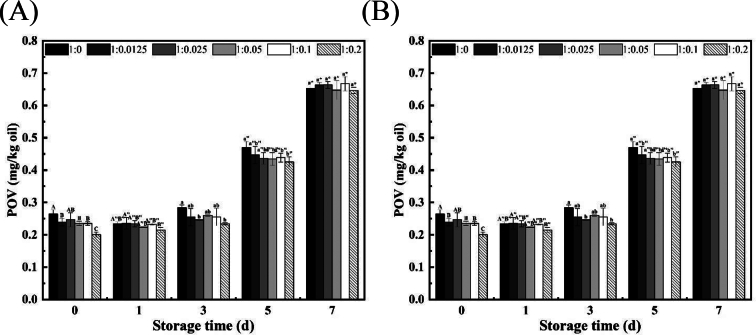
(A) POV of the KO-HIPEs stabilized by SWPs and SWPs-RES complex during storage. (B) TBARS of the KO-HIPEs stabilized by SWPs and SWPs-RES complex during storage.

Antarctic krill oil is known for its high content of unsaturated fatty acids and phospholipids, but its susceptibility to lipid oxidation is a challenge due to the oxidation of double bonds and the hygroscopic nature of phospholipids (Zhao et al., 2020). In order to investigate the oxidation stability of HIPEs of SWPs-RES-KO against lipid oxidation during storage period, the present study was carried out to determine the contents of primary oxidation products and secondary oxidation products stabilized by during storage (40 °C). As shown in Fig. 5A, the POV value of the HIPEs of SWPs-RES-KO gradually in a time-dependent manner and reached highest level on day 7. Different ratios of RES in the HIPEs showed varying effects on POV values, with a ratio of 1:0.2 resulting in the lowest value. Similarly, TBARS values of HIPEs of SWPs-RES-KO increased with storage time, influenced by the RES content (Fig. 5B). Compared to HIPEs of SWPs-KO, those with RES exhibited lower levels of secondary oxidation products, with the 1:0.02 ratio showing the least TBARS value, indicating the antioxidant role of RES during storage. Notably, during days 5–7, HIPEs of SWPs-KO produced more secondary oxidation products than HIPEs of SWPs-RES-KO, highlighting the antioxidant function of RES in lipid oxidation. Furthermore, the stability of the SWP-RES complex at different ratios was considered a crucial factor in the oxidative stability of HIPEs. The stable incorporation of RES within the SWP-RES complex facilitated the consistent antioxidant capability against oxidation phenomena at the oil/water interface in HIPEs, particularly in KO emulsions with elevated phospholipid content. Throughout the storage period of the experiments, all emulsion systems exhibited uniform appearance (data not shown) without significant oil droplet exudation, indicating that all ratios of HIPEs of SWP-RES-KO remained physically stable. Additionally, the interactions between food-derived proteins and polyphenols were believed to be stable during storage, allowing the complex to maintain its position at the oil/water interface due to their inherent hydrophobic properties.

### 3.6 The *in vitro* digestion characteristics of HIPEs of SWPs-RES-KO

**Fig. 6:**
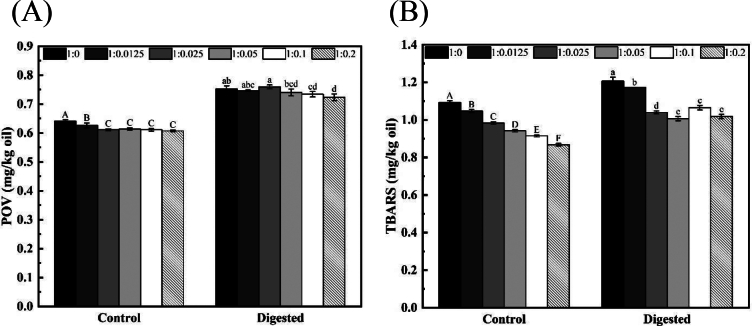
(A) POV of the KO-HIPEs stabilized by SWPs and SWPs-RES complex for *in vitro* digestion. (B) TBARS of the KO-HIPEs stabilized by SWPs and SWPs-RES complex for *in vitro* digestion.

To investigate the oxidation stability of HIPEs of SWPs-RES-KO during human GI digestion, an *in vitro* GI digestion model was established to measure the primary and secondary oxidation products. Fig. 6A&6B illustrated a significant increase in POV and TBARS values in HIPEs of SWPs-KO post-digestion, while HIPEs of SWPs-RES-KO with varying RES additions showed decreasing trends in these values, indicating a dose-dependent antioxidant effect of RES against lipid oxidation during digestion. Among the HIPE samples loaded with SWPs-RES, the 1:0.2 ratio of SWPs-RES-KO exhibited the lowest POV and TBARS values pre- and post-digestion. Notably, all HIPEs of SWPs-RES-KO displayed high levels of lipid secondary oxidation products. Furthermore, HIPEs of Antarctic krill oil stabilized by SWPs-RES complexes exhibited fewer lipid oxidation products post-digestion compared to those stabilized by SWPs alone. The increase in free fatty acids during lipid digestion can lead to further lipid oxidation (Tullberg, Vegarud, & Undeland, 2018), while the addition of RES (a strong antioxidant) to the SWP-RES complex enhanced the antioxidant capacity, contributing to the oxidative stability of HIPEs of SWPs-RES-KO during *in vitro* digestion (Li et al., 2023). On the other hand, the oxygen along with the saliva, chewed food, liquids, and swallowed air could also trigger the lipid oxidation (Kristinova, Storrø, & Rustad, 2013). It was also hypothesized that the use of SWPs-RES could improve the physical stability of HIPEs containing SWPs-RES-KO, particularly when high levels of RES were present. This enhancement might prevent direct oxygen contact by physically isolating the components within the HIPEs (Wang et al., 2023).

## 4 Conclusion

In this study, scallop-derived SWPs successfully bound to RES, forming the SWPs-RES complex. The presence of RES led to changes in secondary structures and fluorescence quenching of the SWPs. Higher amounts of RES enhanced the loading capacity of RES in the SWPs-RES complex, improving antioxidant ability and emulsifying properties of the SWPs. The SWPs-RES complex with higher RES loading showed increased stability in emulsifying properties of HIPEs in SWPs-RES-KO. Additionally, HIPEs of SWPs-RES-OK demonstrated enhanced antioxidant effects against lipid oxidation during storage and *in vitro* digestion. These findings suggest that SWPs-RES complexes are effective emulsifiers and have the potential to enhance the stability of emulsion systems.

## CRediT authorship contribution statement

**Yang Li:** Writing – original draft, Investigation, Data curation. **Xuening Yu:** Data curation. **Rui Liu:** Data curation. **Xiaoyang Liu:** Writing – review & editing, Supervision, Resources, Funding acquisition, Conceptualization. **Fawen Yin:** Data curation. **Deyang Li:** Data curation. **Pengfei Jiang:** Data curation. **Dayong Zhou:** Writing – review & editing, Funding acquisition.

## Declaration of competing interest

The authors declare that they have no known competing financial interests or personal relationships that could have appeared to influence the work reported in this paper.

## Data Availability

The datasets in the current study are available from the corresponding author on reasonable request.

## Appendix A Supplementary data

Supplementary material Image 1
