# Supplementary material for: Application of resveratrol on oxidative stability of protein-based Antarctic krill oil high internal phase emulsion
Source: Food Chem X. 2024 Aug 13;23:101727. doi: 10.1016/j.fochx.2024.101727 (PMC11381618; doi:10.1016/j.fochx.2024.101727)
Supplement: Supplementary file 1 — Supplementary material [file mmc1.docx]

**Supporting Information**

**Application of resveratrol on oxidative stability of protein-based Antarctic krill oil high internal phase emulsion**

Xiaoyang Liu^1,^*, Yang Li^1^, Xuening Yu^1^, Rui Liu^1^, Fawen Yin^1^, Deyang Li^1^, Pengfei Jiang^1^, Dayong Zhou^1^

^1^ SKL of Marine Food Processing & Safety Control, National Engineering Research Center of Seafood, Collaborative Innovation Center of Seafood Deep Processing, Liaoning Province Key Laboratory for Marine Food Science and Technology, School of Food Science and Technology, Dalian Polytechnic University, Dalian 116034, China

*Email: liuxiaoyang0213@126.com.

**Supplementary Figure.1**

**Materials and Methods**

*Determination of particle size of SWPs-RES complexes*

The particle size distribution of the SWPs-RES complexes was measured using a laser particle sizer (90Plus Zeta, Brookhaven, USA) to systematically identify the optimal conditions for complex preparation. The sample solution was diluted 100-fold, and three trials were conducted for the experimental group. All experiments were performed in cuvettes with a width of 1 cm.

**Results**


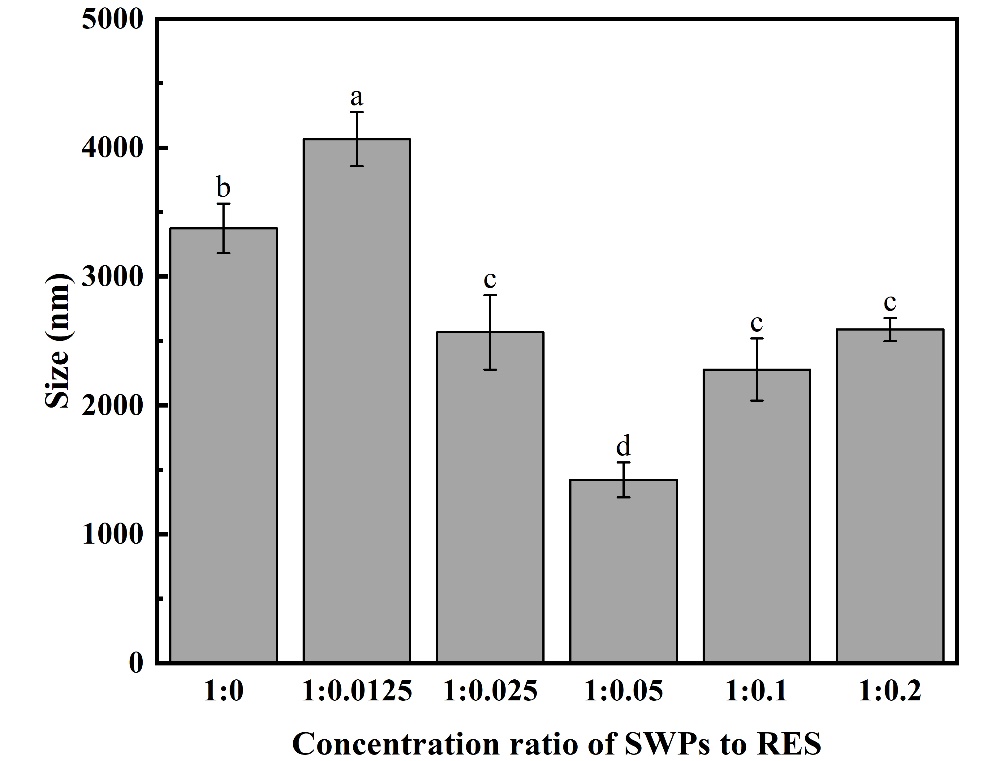


**Figure. S1** Particle size of SWPs-RES complex.

**Supplementary Figure.2**

**Materials and Methods**

*Morphological properties of SWPs-RES complexes by SEM*

The solutions of SWPs-RES complexes in various ratios were freeze-dried, and the resulting powders were subjected to gold-spraying for a few seconds. The morphology of the SWPs-RES complexes was examined using scanning electron microscopy (SEM, Regulus 8100, Hitachi, Japan).

**Results**


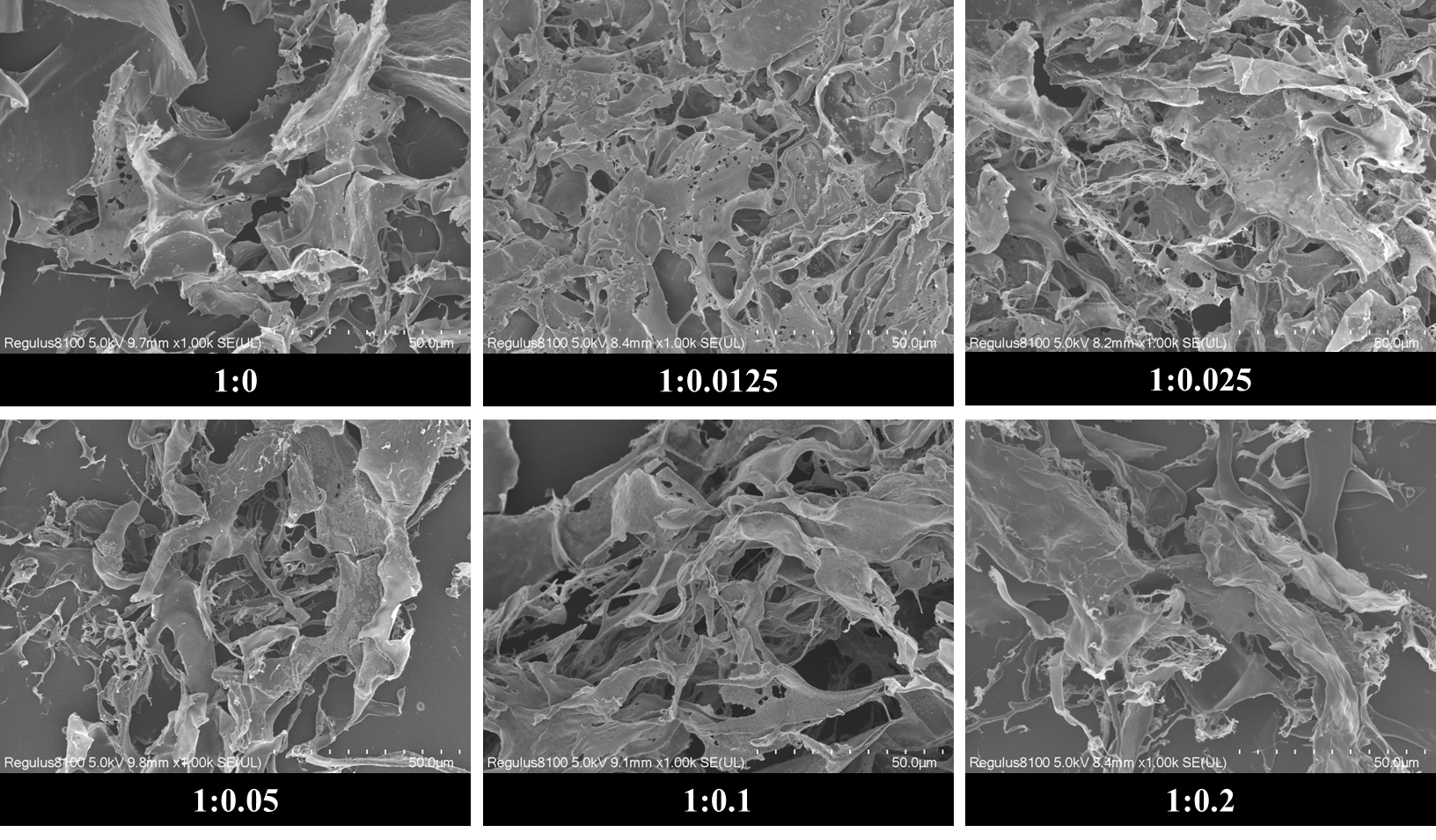


**Figure. S2** SEM images of SWPs-RES complex (х1000).
